# N-docosahexaenoylethanolamine reduces neuroinflammation and cognitive impairment after mild traumatic brain injury in rats

**DOI:** 10.1038/s41598-020-80818-9

**Published:** 2021-01-12

**Authors:** Arina I. Ponomarenko, Anna A. Tyrtyshnaia, Evgeny A. Pislyagin, Inessa V. Dyuizen, Ruslan M. Sultanov, Igor V. Manzhulo

**Affiliations:** 1 A.V. Zhirmunsky National Scientific Center of Marine Biology, Far Eastern Branch, Russian Academy of Sciences, Vladivostok, Russia 690041; 2 G.B. Elyakov Pacific Institute of Bioorganic Chemistry, Far Eastern Branch, Russian Academy of Sciences, Vladivostok, Russia 690022

**Keywords:** Glial biology, Neuroimmunology

## Abstract

At present, there is a growing interest in the study of the neurotropic activity of polyunsaturated fatty acids ethanolamides (N-acylethanolamines). N-docosahexaenoylethanolamine (DHEA, synaptamide) is an endogenous metabolite and structural analogue of anandamide, a widely studied endocannabinoid derived from arachidonic acid. The results of this study demonstrate that DHEA, when administered subcutaneously (10 mg/kg/day, 7 days), promotes cognitive recovery in rats subjected to mild traumatic brain injury (mTBI). In the cerebral cortex of experimental animals, we analyzed the dynamics of Iba-1-positive microglia activity changes and the expression of pro-inflammatory markers (IL1β, IL6, CD86). We used immortalized mouse microglial cells (SIM-A9) to assess the effects of DHEA on LPS-induced cytokines/ROS/NO/nitrite, as well as on CD206 (anti-inflammatory microglia) and the antioxidant enzyme superoxide dismutase (SOD) production. In vivo and in vitro experiments showed that DHEA: (1) improves indicators of anxiety and long-term memory; (2) inhibits the pro-inflammatory microglial cells activity; (3) decrease the level of pro-inflammatory cytokines/ROS/NO/nitrites; (4) increase CD206 and SOD production. In general, the results of this study indicate that DHEA has a complex effect on the neuroinflammation processes, which indicates its high therapeutic potential.

## Introduction

Traumatic brain injury (TBI) is defined as damage to the brain resulting from an external mechanical force, such as rapid acceleration or deceleration, blast wave, compression, penetrating injury, blunt trauma, and can result in temporary or permanent impairment of cognitive, physical and psychosocial functions^1^. According to the clinical studies, 80–90% of all TBI cases are mild (mTBI) but have significant delayed consequences and therefore are a serious problem of modern healthcare. The symptoms of mTBI in the acute phase are usually transient and subtle for the patient himself, however, in some cases, they can be accompanied by a long-term inflammatory process with a delayed outcome in cognitive dysfunction of varying severity^2,3^. Lack of proper and timely therapeutic treatment of mTBI can trigger such pathological conditions and processes as chronic neuroinflammation, oxidative stress, axonal damage, impaired cognitive functions^4,5^.

In most cases, the animal model studies of mTBI are performed by the formation of a closed head injury using a free-falling weight. Such a mechanism for the TBIs formation to the greatest extent imitates the TBIs obtained in real conditions^6^. In this case, the development of a pronounced neuroinflammatory process is observed directly under the site of the injury in the cerebral cortex. The major cell type responsible for the inflammatory response is microglia^7^. Despite the large amount of experimental evidence, the mechanisms regulating microglial polarity in TBI remain unknown. To date, it is known that the activated and reactive microglia states have a dual functional organization, represented by a pro-inflammatory type (classical activation) producing TNFα, IL1β, IL6, nitric oxide (NO) and cell surface markers CD86 and CD68 as well as an anti-inflammatory type (alternative activation) producing IL-4, IL-10 and cell surface marker CD206^8,9^.

In this regard, the effect on microglia using pharmacological agents seems to be the most promising therapeutic option in the treatment of the mTBI consequences. An important direction in this case can be the use of natural compounds derived from marine hydrobionts. A very promising compound is N-docosahexaenoylethanolamine (DHEA, synaptamide), an endogenous metabolite of docosahexaenoic acid (DHA)^10^, which is a structural analogue of N-arachidonylethanolamine (anandamide), a potent arachidonic acid-derived endocannabinoid^11^. Despite its structural similarity to anandamide, synaptamide does not function as an endocannabinoid, mainly due to its weak binding to cannabinoid receptors, but it possesses potent synaptogenic activity, which is why the term “synaptamide” was coined for this metabolite^12^. In recent studies, a number of data have been presented that indicate a high biological activity of DHEA. This substance is able to penetrate the blood–brain barrier (BBB)^13^ and promotes neurogenesis, neurite growth and synaptogenesis in developing neurons^14,15^. DHEA also attenuates the lipopolysaccharide-induced neuroinflammatory response and reduces the deleterious effects of ethanol on neurogenic neural stem cell (NSC) differentiation. These actions are mediated by the specific target receptor for synaptamide GPR110 (ADGRF1), a G-protein coupled receptor. Binding of synaptamide to GPR110 induces cAMP production and phosphorylation of protein kinase A (PKA) and the cAMP reaction binding element protein (CREB). This signaling pathway induce the expression of neurogenic and synaptogenic genes and suppresses the expression of pro-inflammatory genes^16^. In in vitro experiments, synaptamide reduces the LPS-induced inflammatory response, probably by inhibiting the NF-κB factor transcription. The observed effects are similar to the previously established DHA biological activity, however, the activity of synaptamide is at least 10–100 times higher^17,18^.

In this study, we test the hypothesis that DHEA improves cognitive function and mediates neuroprotective activity after mTBI by modulating microglial and antioxidant activity. Animals were subcutaneously injected with DHEA at a dose of 10 mg/kg daily during 7 day after mTBI.

## Results

### Behavioral studies

#### Confirmation of the degree and nature of TBI

The post-sham mortality rate or mTBI was 0%. Differences in recovery time between groups were not observed. All rats regained consciousness after injury in <5 min. The skulls were examined for obvious signs of fracture immediately after each mTBI, and the absence of a skull fracture was confirmed by euthanasia 7 days after the mTBI. The absence of subdural and intracerebral hemorrhages was also noted. The brains of mTBI rats were generally indistinguishable from the brains of sham rats on gross pathological examination. The Neurological Severity Scale (NSS) was used to assess the severity of injury immediately after mTBI induction. There were no significant differences in the distribution of points on the NSS scale in the acute period between animals of all experimental groups (data not shown).

#### Anxiety

**Figure 1:**
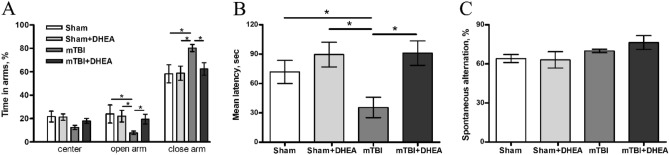
Behavioral effects of mTBI and DHEA treatment. (**A**) Testing anxiety-like behavior with the Elevated plus-maze (EPM). (**B**) The effects of mTBI and DHEA treatment on long-term memory. Passive avoidance test. (**C**) Spontaneous alternation rate differences in Y-maze testing. Data are presented as mean ± SEM, *P < 0.05, n = 14/group (one-way ANOVA, post-test Tukey). Images were prepared with using GIMP 2.10.22 software (http://www.gimp.org/).

Data obtained in the elevated plus-maze (EPM) suggests that mild traumatic brain injury enhances overall anxiety in rats. Animals after mTBI induction spend more time in closed EPM arms (80.35 ± 3.1%) than sham- operated rats (58.22 ± 7.7%). Synaptamide treatment reduced this indicator to values 62.45 ± 5.4% for “mTBI + DHEA” group (Fig. 1A).

#### Long-term memory

Study in the Passive avoidance test showed impairment of long-term memory (24 h) in animals after mTBI. The latency before the dark compartment entering in mTBI-induced rats was significantly reduced compared to DHEA-treated rats (35.5 ± 10.4 s for “mTBI”, 89.56 ± 12.6 s for “Sham + DHEA” group and 91.07 ± 12.5 s for “mTBI + DHEA” group). Sham-operated animals showed a level of long-term memory similar to DHEA-treated (71.86 ± 11.8 s) (Fig. 1B).

#### Working memory

A study in the elevated Y-maze did not reveal significant differences between all experimental groups (Fig. 1C).

### Synaptamide reduces microglial activity in mTBI

The neuroinflammation that occurs in the brain after mTBI develops mainly due to microglial cells activation and the production of pro-inflammatory cytokines. For the immunohistochemical assessment of neuroinflammation severity within the cerebral cortex of experimental rats, the microglial marker iba-1 was chosen. The area of iba-1^+^ staining and the number of immunopositive cells were assessed. The concentration of the iba-1 protein in the cerebral cortex tissue was determined using ELISA.

**Figure 2:**
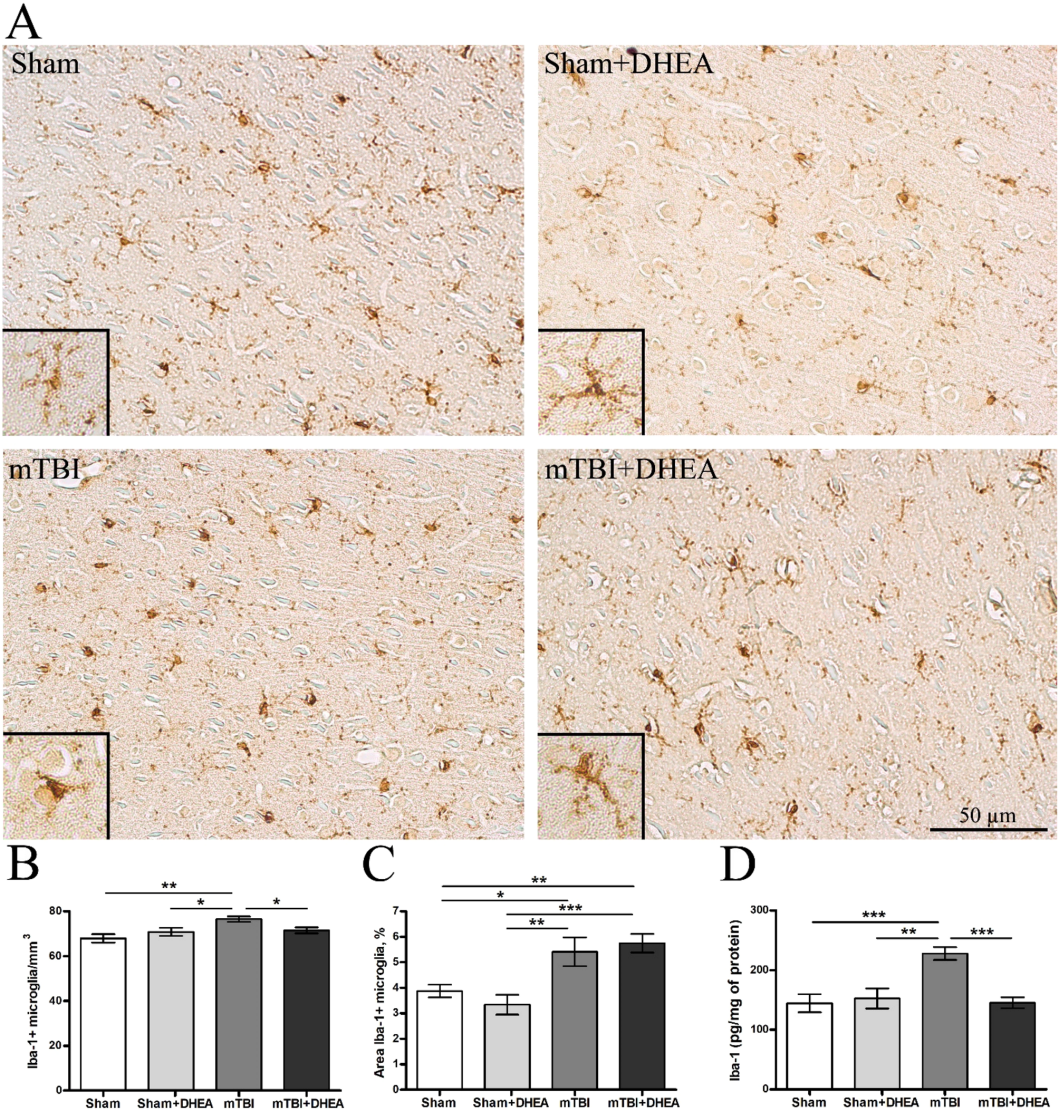
Expression of iba-1-positive microglia in the cerebral cortex in Sham, TBI and after DHEA treatment. (**A**) Representative images of Iba-1 immunohistochemically-stained slides. (**B**) The quantitative distribution of iba-1-positive microglia. (**C**) The staining area of the iba-1-positive microglia in the cerebral cortex. (**D**) The cerebral cortex iba-1 expression. Data are the mean ± SEM, n = 7/group, *P < 0.05, **P < 0.01 and ***P < 0.001 (one-way ANOVA, post-test Tukey). Images were prepared with using GIMP 2.10.22 software (http://www.gimp.org/).

Normally, microglial cells have small cell bodies (9–12 microns) with a narrow rim of the cytoplasm and several thin, long, unbranched processes. Microglia activation by mTBI is characterized by a specific change in cells morphology namely the retraction of processes and the hypertrophy of cell bodies. The cells take on an amoeba shape, which allows them, to migrate into the site of inflammation or injury (Fig. 2A).

Morphometric assessment of the glial reaction dynamics in this case becomes difficult, since the pronounced microglial activity is often accompanied by a decrease in the specific area of distribution (due to the retraction of the processes, rounding of the soma). In our opinion, this is precisely why the morphometric assessment of the iba-1-positive staining area in DHEA-treated animals indicates a more pronounced reaction of microglia cells (Fig. 2C). However, quantitative analysis of iba-1-positive cells showed a decrease in their number after DHEA treatment (Fig. 2B). Using ELISA technique, we found that in mTBI animals, the expression of the iba-1 protein increases sharply compared to the “Sham” group. Treatment of rats with DHEA for 1 week prevented mTBI-induced Iba-1 expression increase (Fig. 2D).

### Synaptamide leads to decreased neuroinflammation in mTBI

**Figure 3:**
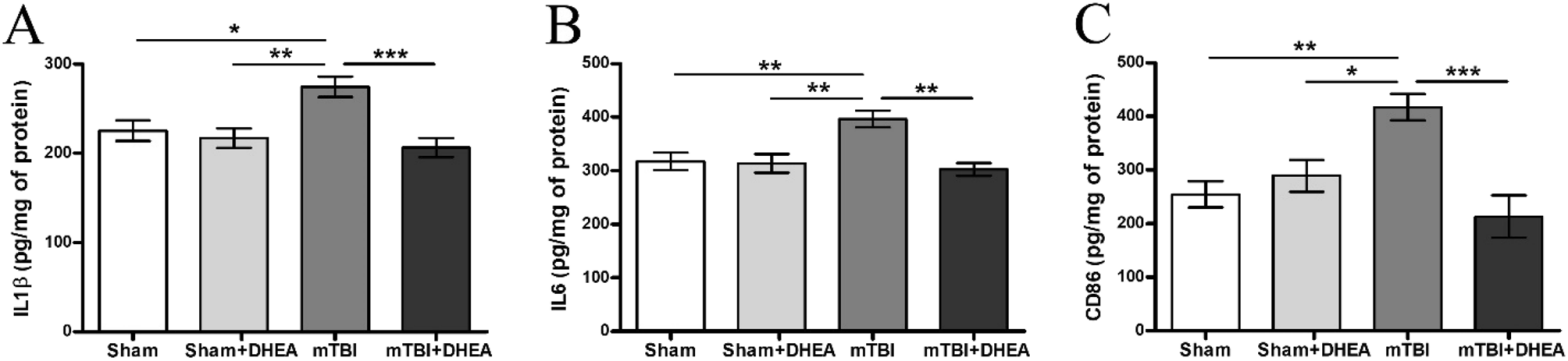
In vivo effect of DHEA on pro-inflammatory responses in mTBI. Level of (**A**) IL1β, (**B**) IL6, (**C**) CD86 expression in the cerebral cortex. Data are the mean ± SEM, n = 7/group, *P < 0.05, **P < 0.01 and ***P < 0.001 (one-way ANOVA, post-test Tukey). Images were prepared with using GIMP 2.10.22 software (http://www.gimp.org/).

The in vivo effect of DHEA on neuroinflammation was tested in rats one week after mTBI. Samples of the cerebral cortex were collected and subjected to ELISA analysis for pro-inflammatory cytokines (IL1β and IL6) and the marker of pro-inflomation activated microglia CD86. In DHEA-treated rats with mTBI, the concentration of IL6 was significantly lower than in the mTBI group (302.4 ± 11.8 pg/mg of protein for “mTBI + DHEA” group and 369.6 ± 15.9 pg/mg of protein for “mTBI” group) (Fig. 3B). In Fig. 3A, we see that trauma significantly increases the IL1β expression level, while DHEA treatment prevented the increase (206.4 ± 10.7 pg/mg of protein for “mTBI + DHEA” group, 274.5 ± 11.3 pg/mg of protein for “mTBI” group and 225.1 ± 11.5 pg/mg of protein for “SHAM” group) (Fig. 3A). A similar situation was observed for CD86 accumulation. In DHEA-treated animals the concentration of CD86 was significantly lower than in non-treated animals (213.7 ± 39.2 pg/mg of protein for “mTBI + DHEA” group and 417 ± 24.9 pg/mg of protein for “mTBI” group) (Fig. 3C).

### In vitro studies

#### Synaptamide has no cytotoxic effect on SIM-A9 microglial cells

Cell viability was determined using flow cytometry following DHEA treatment for 24 h. As shown on Fig. 5E, we did not find a decrease in the synaptamide-treated cells viability for all tested concentrations (Fig. 5E).

#### Synaptamide reverse the LPS-induced microglia morphology changes

**Figure 4:**
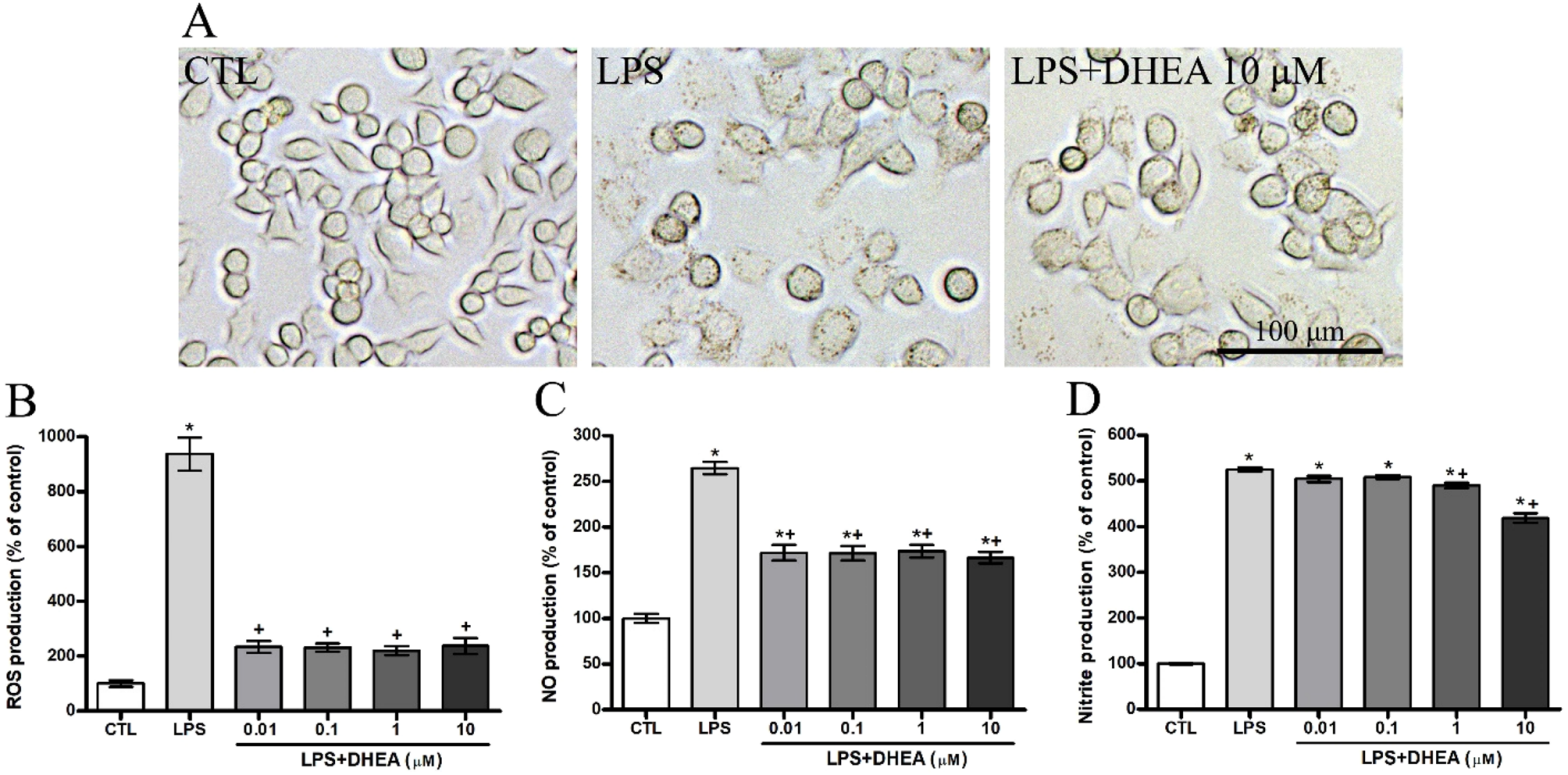
Inhibitory effect of DHEA on LPS-induced production of ROS, NO and nitrites in SIM-A9 microglial cells. (**A**) Representative images of microglial cells exposed to LPS and DHEA. Histogram showing the level of (**B**) ROS, (**C**) NO, (**D**) nitrites production in LPS-activated microglia cells treated with different concentrations of DHEA. *Significant differences between “CTL” and “LPS”, “LPS + DHEA” at similar observation points. ^+^Significant differences between “LPS” and “LPS + DHEA” at similar observation points. Data are the mean ± SEM, n = 9 (number of analyzed samples), *P* < 0.05, (one-way ANOVA, post-test Tukey). Images were prepared with using GIMP 2.10.22 software (http://www.gimp.org/).

We found microglial morphology changes in LPS-treated cells. In Fig. 4A we demonstrate the cells in the control wells with a rounded and fusiform shape with a smooth surface. After 24-h incubation with LPS, microglial cells become more spread and increase in size, which indicates their activation. Synaptamide at a concentration of 10 μM reduces the morphological signs of activation. The number of granules in the cells decreases, the surface smoothed out and the size returns to the control values.

#### Anti-inflammatory effect of synaptamide on LPS-induced microglia cells

To study DHEA anti-inflammatory activity microglial cell culture SIM-A9 were exposed to different DHEA concentrations and subsequently activated by 1 μg/ml LPS solution. Incubation with DHEA resulted in diminished levels of reactive oxygen species (ROS) (Fig. 4B), NO (Fig. 4C) and nitrite production level (Fig. 4D). The results of ELISA carried indicate a significant increase in ROS, NO and nitrites production after LPS treatment.

DHEA in all concentrations reduced the ROS level by several times (from 937.2 ± 59.89% for LPS to 233.3 ± 21.47% for DHEA 0.01 μM). Endogenous NO production is also reduced by synaptamide, regardless of concentration (from 246.5 ± 6.7% for LPS to 171.9 ± 8.6% for DHEA 0.01 μM). The Griess method showed a significant decrease in nitrite production only for concentrations of 1 and 10 μM compared to the level of LPS-activated cells (from 524.9 ± 4.2% for LPS to 490.1 ± 6.2% for DHEA 1 μM and 418.7 ± 10.46% for DHEA 10 μM).

**Figure 5:**
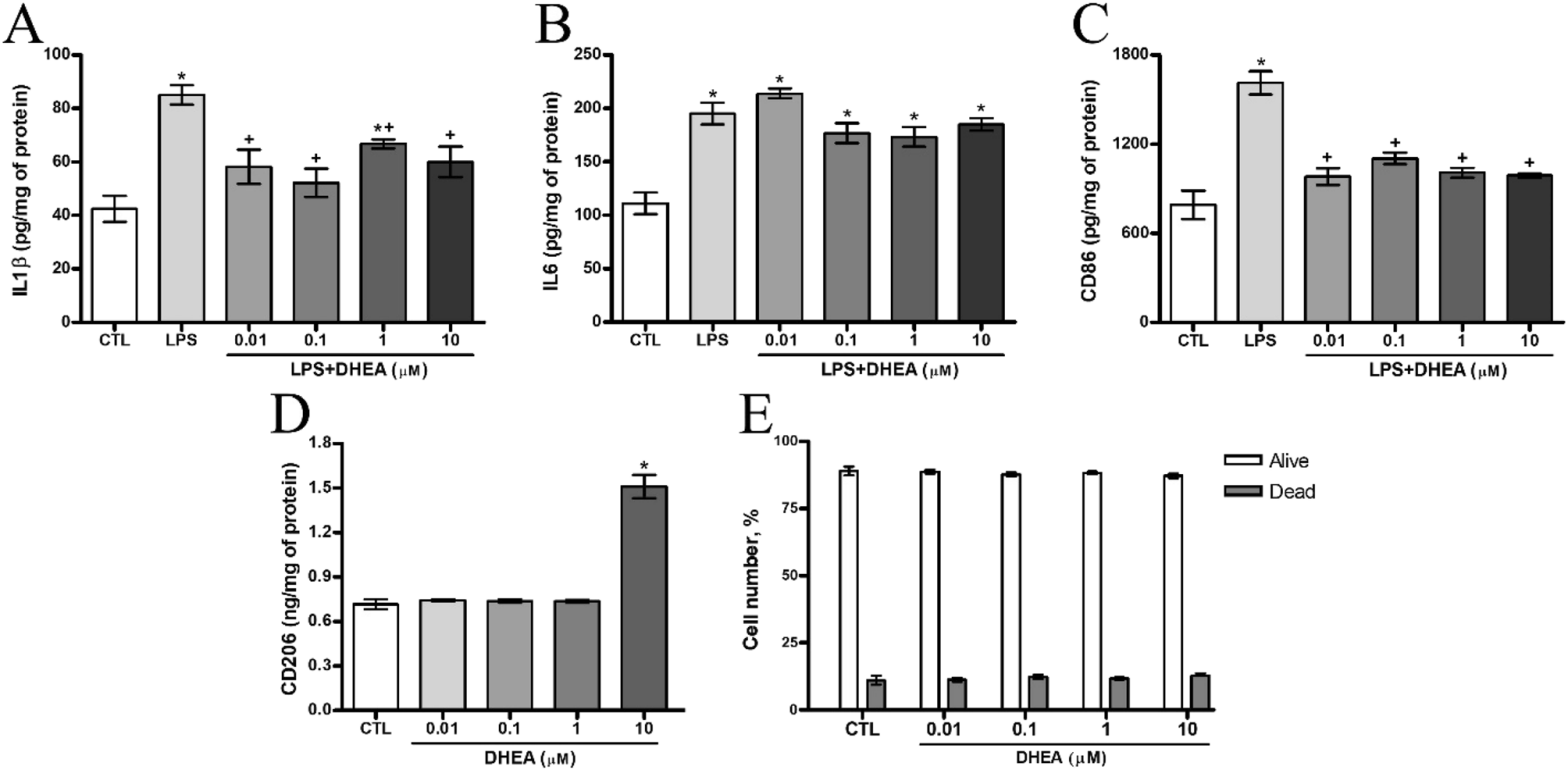
Suppression of pro-inflammatory responses by DHEA in LPS-activated SIM-A9 microglial cells. Level of (**A**) IL-1β, (**B**) IL6, (**C**) CD86, (**D**) CD206 expression in the microglial cells. (**E**) A histogram demonstrating the absence of DHEA effect on apoptosis of microglia cells. *Significant differences between “CTL” and “LPS”, “LPS + DHEA” at similar observation points. ^+^Significant differences between “LPS” and “LPS + DHEA” at similar observation points. Data are the mean ± SEM, n = 9 (number of analyzed samples), *P* < 0.05, (one-way ANOVA, post-test Tukey). Images were prepared with using GIMP 2.10.22 software (http://www.gimp.org/).

To characterize the synaptamide effects on neuroinflamation, the LPS-induced IL1β and IL6 production was measured using ELISA. LPS treatment significantly increased the expression of these cytokines compared to the control level (Fig. 5A, B). After treatment with 0.01, 0.1 and 10 μM synaptamide, LPS-induced IL1β expression decreased from 85.03 ± 3.6 to 58.16 ± 6.3, 52.16 ± 5.3 and 60 ± 5.7 pg/mg of protein respectively (Fig. 5A). In spite of this, the data of ELISA analysis did not show significant differences in the expression of IL6 after treatment with DHEA in LPS-stimulated microglial cells (Fig. 5B).

The expression of the pro-inflammatory microglial marker CD86 by cells also increased upon LPS treatment. Treatment with DHEA at a concentration range from 0.01 to 10 μM reduced the expression level to the control level (Fig. 5C). On the contrary, the expression of CD206 (marker of anti-inflammatory microglia) increased in comparison with the control level when the cells were treated with DHEA at a concentration of 10 μM (Fig. 5D).

#### Synaptamide improves the antioxidant activity in microglia cells

**Figure 6:**
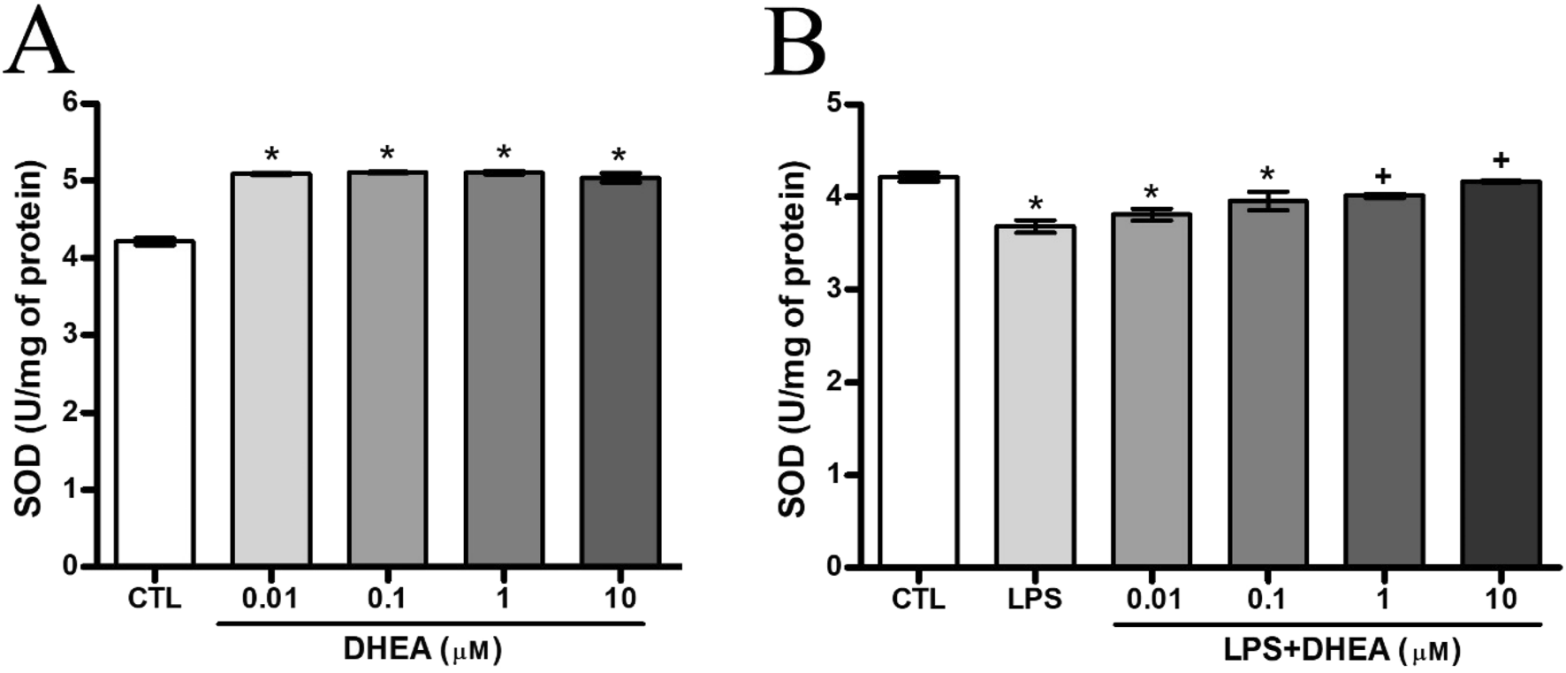
DHEA increases the activity of SOD (**A**) and during LPS pretreatment (**B**). *Significant differences between “CTL” and “LPS”, “LPS + DHEA” at similar observation points. ^+^Significant differences between “LPS” and “LPS + DHEA” at similar observation points. Data are the mean ± SEM, n = 9 (number of analyzed samples), *P* < 0.05, (one-way ANOVA, post-test Tukey). Images were prepared with using GIMP 2.10.22 software (http://www.gimp.org/).

Antioxidant activity was assessed both on DHEA- and LPS-treated SIM-A9 microglial cells. DHEA treatment in all studied concentrations increased SOD activity equally and therefore increased the antioxidant defense of cells (4.21 ± 0.05 U/mg for control and 5 ± 0.06 U/mg of protein for cells treated with DHEA at a concentration of 10 μM) (Fig. 6A). The addition of LPS to the culture medium significantly reduced the enzyme expression (3.68 ± 0.07 U/mg of protein). Treatment with DHEA in dosages from 1 to 10 μM restored SOD to almost control values (Fig. 6B).

## Discussion

The results of the present study indicate that the use of DHEA leads to the development of several metabolic and cellular events that contribute to the cognitive functions recovery in animals with mTBI. To simulate the injury, a weight-drop model was used with a rod weighing 337 g falling from a height of 8 cm onto a previously scalped rat skull. After injury, each animal was examined for a skull fracture; animals with open TBI were excluded from the experiment. Immediately after injury, each animal received the first injection of DHEA (dose 10 mg/kg). In order to determine the severity of the injury and to exclude animals subjected to a more severe TBI, the animals' general neurological and motor functions were assessed using the NSS scale 1 h after the surgery^19^. According to the results of the NSS tests, no animals were removed from further experiment.

Mild TBI is most characterized by the absence of structural changes on conventional imaging, but by the presence of cognitive dysfunction. While most patients recover within days or weeks of a concussion, some people develop long-term or progressively worsening symptoms^20^. It was previously shown that mTBI increases the general level of anxiety in rodents^21^. In our experiment, the cognitive functions of experimental animals were assessed by three parameters: (1) anxiety levels, (2) long-term and (3) working memory state. The drug exhibited anxiolytic activity, reducing the level of anxiety. Animals from the “mTBI + DHEA” group spent much less time in the closed arms of the elevated plus-maze. In our study, the passive avoidance test was used to assess long-term memory. The memory deterioration was observed 7 days after mTBI induction. Despite the stressful experience, animals after mTBI re-entered the dark compartment. At the same time, in animals of the “Sham” group and DHEA-treated rats, the average latency before entering the dark section was significantly higher than in the “mTBI” group. Thus, DHEA reversed the mTBI-induced cognitive deficits. No impairment of working memory was observed in experimental animals. It can be assumed that a 7 days period after mTBI induction is not enough to impair this type of memory. Previous studies show that chronic loss of working memory developed in 8 weeks after TBI^22^ and continued up to 4 months^23^.

In TBI, both microglia and astroglia are involved in the inflammatory and reconstructive processes regulation in the area of injury^24^. The pathological immune response is one of the most significant destructive processes after TBI. It is the regulation of the immune response realized by the specific microglial activity that can become an important therapeutic option in the TBI treatment. In TBI, the immune response is multidirectional and is mediated by different activities of pro-inflammatory and anti-inflammatory types of microglia^25^. First type of microglia produces pro-inflammatory cytokines (IL1β, IL-6, TNFα), chemokines (CCL2, CXCL9, CXCL10), NO and ROS, which perform protective functions in the acute period after injury^26,27^. However, dysregulated, or excessive pro-inflammatory processes in the brain can cause neurotoxicity due to the release of pro-inflammatory factors and neurotoxic mediators that trigger vicious cycles of microglia-mediated neurodegeneration^28^. During activation, microglia morphology transforms from branched to amoeboid form, and cells secrete pro-inflammatory cytokines and free radicals that are cytotoxic to neurons and can contribute to the process of neurodegeneration after TBI^29^. The development of neurodegenerative diseases and impaired cognitive functions in humans after single or multiple cases of mTBI is associated precisely with excessive neuroinflammation. Studies of human brain samples after mTBI have shown that reactive microglia are present in tissue up to 18 weeks after trauma^30^. That is why it is important to consider the time course of microglia activation along these pathways. In our study, 7 days after mTBI, an inflammatory process is also observed in the ipsilateral cerebral cortex, which is accompanied by an increase in the number of microglia and the formation of an amoeboid morphological state. DHEA administration reduced the number of iba-1-positive microglia to the level of sham-operated animals. At the same time, the increase in the area of microglia staining is probably associated with the predominance of the anti-inflammatory reactive microglia population. Previous studies have already described the special morphological state of microgliocytes—the so-called reactive microglial cells, the morphology of which differs from the above-described resting and activated cells^31^. Reactive microgliocytes are rod-like cells with long branching processes having numerous varicose dilatations. This cell type is often found in areas of axonal or dendritic degeneration, where they transform into reactive macrophages exhibiting phagocytic activity^32^. In our study, ELISA revealed an increase in the pro-inflammatory cytokines (IL1β, IL6), as well as in the inflammatory-activated microglia cell surface marker CD86 production within the cerebral cortex on the 7th day after mTBI. Thus, daily administration of DHEA to animals with mTBI reduced the level of both Iba-1 + -microglia and the pro-inflammatory microglia’s markers including IL1β, IL6 and CD86.

The results of in vitro experiments demonstrated an increase in the microglial marker CD206 level, which indicates anti-inflammatory cell activation, and an increase in the superoxide dismutase production in microglial cells after DHEA treatment. This further emphasizes the compound's anti-inflammatory activity, which is likely to underlie the cognitive improvement in experimental animals.

As noted earlier, pro-inflammatory microglial activation is usually accompanied by morphological changes, an increase in the pro-inflammatory cytokines synthesis, and the release of NO, ROS and free radicals. DHEA treatment of microglial cell culture prevented the development of all the above-mentioned LPS-mediated pathological effects of neuroinflammation. The ability of DHEA to reduce the level of LPS-induced pro-inflammatory activity has already been described in the literature^33^, but our data significantly expand the spectrum of the observed effects. In our in vitro study using a model in which cells are first incubated for 1 h with DHEA (concentration range from 0.01 to 10 μM) and only then are activated by LPS (1 μg/ml), we showed a decrease in LPS-induced inflammation by inhibition of the ROS, NO, nitrite, IL1β and cell surface microglial marker CD86 production, as well as an increase in the SOD synthesis. It is known that DHEA effectively inhibits LPS-induced expression of pro-inflammatory cytokines by enhancing cAMP/PKA signaling, as well as suppressing the nuclear translocation of NF-κB p65^33^.

The results of this study indicate that DHEA has a complex effect on the course of the recovery process after mTBI, which indicates its high therapeutic potential. The mechanisms of the DHEA anti-inflammatory and neuroprotective activity require additional detailed studies that can ensure its introduction into clinical practice for the central nervous system pathologies treatment.

## Materials and methods

### N-Docosahexaenoylethanolamine

The concentrate of polyunsaturated fatty acids was obtained according to the method Ermolenko et al.^34^ from squid Berryteuthis magister liver. Then concentrate of polyunsaturated fatty acids was converted to ethyl esters and treated with ethanolamine to obtain ethanolamines of fatty acids (United States, Patent 3,257,436). HPLC of polyunsaturated fatty acid ethanolamines was performed on a Shimadzu LC-8A chromatograph (Shimadzu, Japan) with UV/VIS SPD-20A (205 nm). Separation was carried out on a preparative reverse-phase column Supelco Discovery HS C-18 (Bellefonte, PA); 10 µm particle size, 250 mm × 50 mm i.d. Isocratic elution with the system ethanol/water (70:30, v/v) was used. The elution rate was 50 ml/min. Fractions containing DHEA was collected, evaporated under vacuum and analyzed by GC and GC–MS. DHEA was 99.4% purity and was fluid light-yellow oil with an unexpressed smell at room temperature.

### Animals

All procedures were approved by the Animal Ethics Committee at A.V. Zhirmunsky National Scientific Center of Marine Biology, Far Eastern Branch of the Russian Academy of Sciences, Vladivostok, Russia, in accordance with the Guidelines for the protection of the health of laboratory animals. Male Wistar rats (260 ± 20 g, age 3 months), 14 in each group (“Sham”, “Sham + DHEA”, “mTBI”, “mTBI + DHEA”) were used for experiments. The rats were kept 2–4 in a cage with unlimited access to food and water. The animals were maintained at constant temperature (23 ± 2 °C) and humidity (55 ± 15%) with a 12-h light/dark cycle (light at 7:00 AM). The rats were handled for 5 min once a day for 5 days before the experiments.

### mTBI procedure and treatments

**Figure 7:**
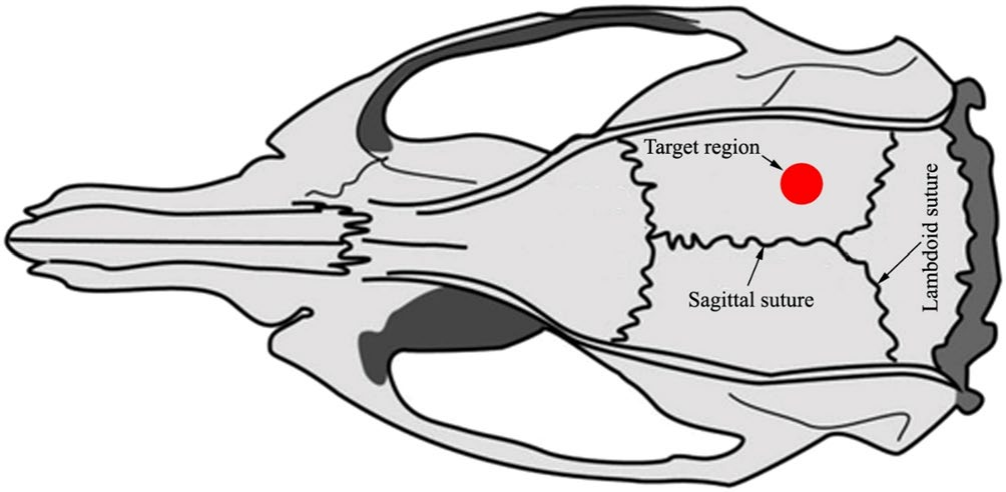
Anatomical mapping and target area for rod impact. The blow was dealt to the right hemisphere 2 mm lateral from the midline and 4 mm forth from the lambdoid suture. The red dot indicates the place of application of force. Institutional animal care committee permission was obtained for all experiments. Images were prepared with using GIMP 2.10.22 software (http://www.gimp.org/).

Initially the animals were divided into 4 groups: the “Sham” group (n = 14); group “Sham + DHEA” (n = 14)—animals injected with DHEA; group “mTBI” (n = 14)—animals with mild traumatic brain injury; group “mTBI + DHEA” (n = 14)—animals injected with DHEA, with mild traumatic brain injury. After general anesthesia with 4.5% Isofluorane in 100% oxygen (Anesthesia Systems (Harvard Apparatus)) for approximately 1–2 min, maintained through a nose cone to minimise rats suffering during the procedure, as described previously^35^. A midline incision over the skull was performed, the skin retracted, and the skull exposed to locate the area of impact. The head was manually fixed on the bottom platform of the weight-drop device (2700, Northeast Biomedical Inc., MA, USA). Metal rod weighting 337 g with a blunt tip of 3 mm diameter was gently advanced onto exposed rat skull at the right hemisphere 2 mm lateral from the midline and 4 mm forth from the lambdoid suture (Fig. 7). The head was held in place manually while the rod was uplifted at the 8 cm height and held by a lever. The trauma was performed by lowering the lever and the rod free-drop onto the rat skull^19^. Animals were observed for seizures (not observed) and overt signs of skull fracture after mTBI (not observed) were assessed. In the sham groups, the animal underwent a surgical operation identical to the one described above, with the exception of the simulation of mTBI. After surgery, the skin was sutured and treated with an antibacterial spray to prevent infection. The animals were immediately injected subcutaneously with DHEA or saline. They were left to recover on a warm pad until thermoregulation and an alert state was reestablished. They were then returned to their home cages, with free access to food and water. Emulsion of DHEA was prepared by dissolving in 0.9% saline. Animals were subcutaneously injected with DHEA emulsion at a dose of 10 mg/kg daily during 7 day after mTBI. Animals in the “Sham” and “mTBI” groups were injected with 0.9% saline in the same mode. DHEA dosage was chosen using the literature data and our previously obtained results. To date, there is no data on the use of DHEA in TBI, however, it is known that DHEA at a concentration of 10 mg/kg has an antinociceptive effect in mice^36,37^. In addition, in previous DHEA studies in a model of LPS-induced inflammation in mice a dosage of 2 to 10 mg/kg was used^33,38^.

### Behavioral testing

All behavioral tests were performed during the light cycle between 7:00 AM and 7:00 PM. To minimize olfactory signals, the apparatus was thoroughly cleaned with 70% ethanol after each animal. On the day of testing, the rat was left in its home cage used for the experiment for 2 h before the start of the behavioral study. Assessment of neurological functions using the NSS scale was performed 1 h after the surgery. Other behavioral tests were performed from 5 to 7 days after mTBI, to eliminate the potential impact of animals’ over-testing on the results. Working memory was tested on day 5 after mTBI, anxiety-like behavior was studied on day 6 in the morning, and the learning phase of the passive avoidance test was studied on the same day in the evening. On day 7 before sacrifice, a passive avoidance test session was performed.

#### Assessment of neurological function

Gross neurological motor function was evaluated using a neurological severity score^37^. The NSS is derived from a 9‐item checklist assessing: monoparesis/hemiparesis, exit circle, startle reflex, inability to walk straight and seeking behavior. Additionally, functional tests assessed: failure to balance for 30 s on round beam, and inability to cross a beam (1, 2 and 3 cm) without exhibiting foot faults. Investigators were blinded to the injury status of each animal and gave each animal a score of either 1 (yes) or 0 (no) on each assessment, summing all scores for a maximum possible NSS of 9, such that scores increased with severity of dysfunction. No animals were excluded from analysis.

#### Working memory

To assess working memory, a Y-maze spontaneous alternation test was performed. The Y-maze, made of opaque acrylic glass, consisted of 3 equal arms (length: 50 cm, width: 15 cm, height: 25 cm). The animals were alternately placed in the center of the maze and left to move freely for 5 min. To minimize audible cues, camera observation was carried out by the operator sitting in an adjoining room. Arm entry was assessed when the rat entered the arm with all four paws. The total number of entries into hands (N) and the number of “correct” triplets (M, sequential selection of each of the 3 hands without repeated entries) were evaluated. The alternation rate was calculated using the formula: R(%) = M × 100/(N–2)^39^. Rats with normal working memory tend to enter different arms of the maze, i.e. the frequency of their spontaneous alternation is higher than^40^.

#### Elevated plus-maze (EPM)

The EPM is a gold standard for testing anxiety-like behavior in rodents^41^. The elevated plus maze (Panlab/Harvard Apparatus, USA) was an apparatus consisted of two open arms (50 × 14 cm, surrounded by a 1 cm-high border) and two closed arms (50 × 14 cm, surrounded by 30 cm-high walls), with the two pairs of identical arms, which emerged from a central platform (14 × 14 cm), positioned opposite each other. Each rat was placed in the center of the maze with the head pointing toward the o arms, and was then allowed to move freely for 5 min. Video system SMART 3.0 (Panlab/Harvard Apparatus, USA) software was used to quantify behavior. The center of the mouse was tracked, and time spent in open, closed arms and central zone was recorded.

In the EPM, the following parameters were measured: time spent in the open arms, closed arms, and central platform, head dipping and risk assessments (the latter two are not reported, as no effects were seen).

#### Passive avoidance test

The effects of mild traumatic brain injury and DHEA treatment on long-term memory was evaluated using a passive avoidance test^42^. The testing apparatus consisted of light and dark compartments separated by a sliding door (Panlab Harvard Apparatus, USA). In the training session, the rat was placed in the light compartment and allowed to explore for 60 s before the sliding door was opened. When the animal entered the dark compartment, the door was closed and 2 s later, an inescapable electric foot-shock (0.3 mA, 2 s) was delivered. The test session was performed 24 h after the training session without the electric foot-shock. The step-through latency for animals to enter the dark compartment was measured.

### Immunohistochemistry and microscopy

Brain tissue was harvested 7 days post mTBI, rats (n = 7 animals/group) were anesthetized with 4.5% isofluorane in 100% oxygen (Anesthesia Systems, Harvard Apparatus, USA) and transcardially perfused with 20 ml of ice-cold saline followed by 20 ml of cold fixative (4% paraformaldehyde in 0.1 M phosphate buffer (PBS), pH 7.2). The cranium was then immediately opened, the brain removed and then fixed for 24 h at 4 °C in fresh buffered 4% paraformaldehyde. After washing in PBS, the brain was processed and embedded in paraffin according to standard embedding methods. Paraffin sections (7 μm) were obtained from the brain segment located immediately below the target area (Bregma −4.56 mm). After dewaxing, they were placed in H_2_O_2_ (3%) for 10 min and incubated in a blocking buffer containing 2% bovine serum albumin (SC-2323, Santa Cruz, USA) and 0.25% Triton X-100 (Gerbu, USA) in within 1 h. Incubation with primary rabbit antibodies: iba-1, 1:1000 (ab178846, Abcam, USA) was carried out overnight at 4 °C. A negative control (without primary antibody) was also performed. Appropriate secondary antibodies conjugated to horseradish peroxidase (PI-1000, anti-rabbit) were used according to the manufacturer's instructions (Vector Laboratories, USA). After washing, sections were treated with chromogen (TL-060-QHD, Thermo Scientific, USA) for 5–10 min to elicit the immunoperoxidase reaction. The sections were then washed with distilled water, dehydrated and mounted onto slides using mounting medium (CS705, Dako, USA).

**Figure 8:**
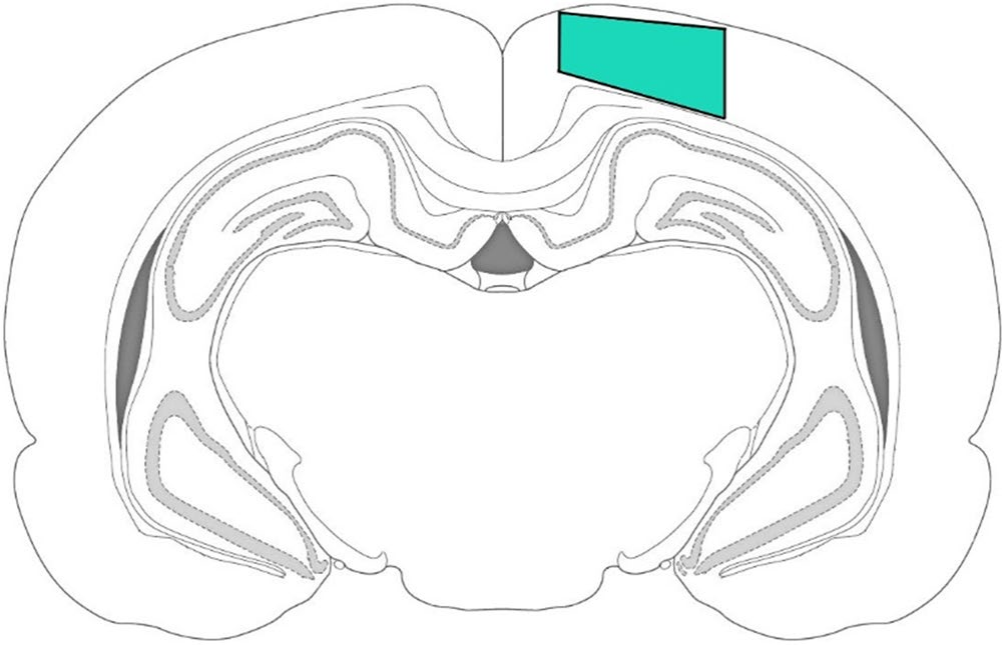
The approximate location of the ipsilateral cerebral cortex region where images for microglia quantification were taken (marked in green). Images were prepared with using GIMP 2.10.22 software (http://www.gimp.org/).

Images were acquired at a size of 530 × 710 μm with a ×20 NA 0.45 dry objective lens (Plan-Apochromat) on a microscope (Axio Image Z2) equipped with a CCD camera (AxioCam HRc) (Carl Zeiss, Germany). For analysis, at least 70 images ipsilateral cerebral cortex were used for each group of animals, at least two images from one slice of the brain (Fig. 8). The number and staining area of the iba-1-positive microglia were determined in each 10th section of the brain using ImageJ software (NIH, USA). The number of iba-1-positive cells/mm^3^ was calculated by the formula: d = (10^6^ × n)/(S × 7), where d—equals cell density; 10^6^—the coefficient converting μm^2^ into mm^2^; n—the number of immunopositive cells; S—area of the cerebral cortex (μm^2^); and 7—slice thickness (μm). The ratio of the area of the studied region of the cerebral cortex to the area of immunopositive staining was expressed in percent. All measurements were performed by an operator blinded to the sections’ identity.

### Cells for bioassay

The murine microglial cell line SIM-A9 (CRL-3265, ATCC, UK) obtained from the American Type Culture Collection was used. Cells were cultured in standard DMEM medium containing 10% FBS, 5% DHS and 0.5% penicillin/streptomycin at 37 °C in a humidified atmosphere with 5% CO_2_. For determination of ROS, NO, nitrites, SIM-A9 murine microglia were plated into 96-well microplates (1 × 10^3^ cells/well), for determination of SOD and ELISA cell were plated into 24-well plates (1 × 10^5^ cells/well) and incubated at 37 °C with 5% CO_2_ for 1 h. After adhesion the cultural medium was replaced by a medium containing solution DHEA (0.01, 0.1, 1, 10 μM). Cells were cultured for 1 h, then LPS (1 μg/ml) was added and incubated for 24 h Cells incubated in normal medium and cells incubated in medium with LPS but no DHEA were used for negative and positive controls, respectively. Each experiment in vitro was performed independently at least three times.

### Flow cytometry

SIM-A9 murine microglia were plated into 24-well microplates and cultured in complete DMEM/F12 media and incubated at 37 °C with 5% CO_2_ for 1 h. After adhesion the cultural medium was replaced by a medium containing solution DHEA (0.01, 0.1, 1, 10 μM). Cells were cultured this way at 37 °C with 5% CO_2_ for 24 h. Cells incubated in a normal culture medium without DHEA were used as negative controls. To assess the level of apoptosis in SIM-A9 murine microglia cells we used method was based on the use of fluorescent dye propidium iodide (PI, P4864, Sigma-Aldrich, USA). PI does not have any specific carriers integrated into the membrane and is able to penetrate into the cytoplasm and nucleus only through damaged membranes; this typically is able to take place in the final stages of apoptosis during the formation of apoptotic bodies or cell necrosis^43^. Thus, living cells in the sample were not stained by PI.

Prior to the staining, the cells were treated using enzymatic dissociation by TrypLE (12604–021, Gibco, USA) at 37 °C for 5 min, followed by centrifugation (120×*g* for 3 min, at 20 °C). Cell were collected for staining in 12 × 75 mm cytometric tubes (110410, Globe Scientific, Paramus, NJ, USA). A total of 1 µl of propidium iodide solution was added to 100 µl of the cell suspension (1 × 10^6^ cells/ml) with a final concentration of 1 µg/ml. Samples were incubated with PI for 10 min in the dark, RT, then washed once with PBS solution. Samples were analyzed by a flow cytometer (CytoFlex, Beckman Coulter, Brea, CA, USA). At least 10,000 single cells were analyzed for each sample. Cell aggregates were discriminated from the analysis. Analysis of the results was performed using Kaluza software (Version 1.3, Beckman Coulter, Brea, CA, USA).

### Nitric oxide

After 24-h incubation of microglia cells with LPS and DHEA, the endogenously generated NO detection was performed. Cells were coincubated with 10 μM DAF-FM diacetate (D23844, Thermo Fisher Scientific, USA) for 40 min at 37 °C. Prior to detection of fluorescence, cells were washed three times with PBS. The fluorescence intensity was measured using a PHERAstar FS plate reader (BMG Labtech GmbH, Ortenberg, Germany). Green fluorescence of cells was recorded at λ_ex_ = 460 nm and λem = 524 nm. Results are presented as percentage of negative controls.

### Griess

After incubating for 24 h nitrite accumulated in the culture medium was measured as an indicator of NO production using the Griess method^44^. Briefly, 100 μl of the cell culture medium were mixed with 100 μl of Griess reagents and incubated at room temperature for 10 min and the absorbance was determined at 540 nm with an iMark microplate absorbance reader (Bio-Rad, USA). The results were presented as a percentage of negative control.

### Reactive oxygen species

Microglial cells were incubated in a medium containing different concentrations of LPS and DHEA for 24 h. For level analysis ROS formation, 20 µl of 2,7-dichlorodihydrofluorescein diacetate solution (Molecular Probes, Eugene, OR, USA) was added to each well, such that the final concentration was 10 mM, and the plate was incubated for 10 min at 37 °C. The intensity of dichlorofluorescein fluorescence was measured with plate reader PHERAstar FS (BMG Labtech, Ortenberg, Germany) at λ_ex_ = 485 nm, and λ_em_ = 518 nm. The data were processed by MARS Data Analysis V.3.01R2 (BMG Labtech, Ortenberg, Germany). The results were presented as a percentage of negative control.

### ELISA

To quantify the concentration of iba-1, IL1β, IL6, CD86 and CD206 in the rat cerebral cortex and SIM-A9 murine microglia cell, an enzyme-linked immunosorbent assay was obtained. Ipsilateral cerebral cortex harvested 7 days after mTBI (n = 7 animals/group) and treated cells were homogenized on ice in the extraction buffer (100 mM Tris, pH 7.4, 150 mM NaCl, 1 mM EGTA, 1 mM EDTA, 1% Triton X- 100, and 0.5% sodium deoxycholate), with 1 mg/ml of protease inhibitor cocktail (Complete; Sigma-Aldrich, USA) and 0.01 mg/ml phosphatase inhibitor cocktail (P5726; Sigma-Aldrich). The rat AIF1/IBA1 ELISA kit (LS-F8398, LSBio, USA), rat CD86 ELISA kit (RAB0887, Sigma-Aldrich, USA), rat IL-6 ELISA Kit (ab234570, Abcam, USA) and the rat IL-1β ELISA Kit (ab255730, Abcam, USA) were used to measure protein concentrations in cerebral cortex. The mouse IL-1β ELISA Kit (ab197742, Abcam, USA), mouse IL-6 ELISA Kit (ab100713, Abcam, USA), mouse CD86 ELISA Kit (LS-F15288, LSBio, USA) and the mouse CD206 ELISA Kit (LS-F7148, LSBio, USA) were used to measure protein concentrations in microglia cells. BCA protein assay kit (Pierce, Rockford, IL, USA) was used to determine total protein concentrations. Absorbance at 450 nm was measured with an iMark microplate absorbance reader (Bio-Rad, USA).

### Evaluation of SOD enzyme activities

After coincubation with DHEA and LPS in a 24 h total SOD activity was carried out in cell supernatants using the commercial kit (19160, Sigma Aldrich, USA), and calculated as U/1 mg of protein. The calibration curve was constructed using superoxide dismutase (S9697, Sigma Aldrich, USA).

### Statistical analysis

Data were subjected to statistical analysis using one-way ANOVA tests followed by a post hoc Tukey’s multiple comparison test. Data were shown as mean ± SEM, and p < 0.05 was regarded as statistically significant^45^. All statistical tests were performed using the GraphPad Prism 4.00 software (GraphPad Software, San Diego, CA, USA).

## Data availability

The datasets used and analyzed during the current study are available from the corresponding author on reasonable request.
